# Flow virometry analysis of envelope glycoprotein conformations on individual HIV virions

**DOI:** 10.1038/s41598-017-00935-w

**Published:** 2017-04-19

**Authors:** Anush Arakelyan, Wendy Fitzgerald, Deborah F. King, Paul Rogers, Hannah M. Cheeseman, Jean-Charles Grivel, Robin J. Shattock, Leonid Margolis

**Affiliations:** 1 Section of Intercellular Interactions, Eunice Kennedy Shriver National Institute of Child Health and Human Development, National Institutes of Health, Bethesda, Maryland USA; 2 Mucosal Infection & Immunity Group, Department of Medicine, Imperial College, London, UK; 3 Sidra Medical and Research Center, Doha, Qatar

## Abstract

HIV-1 envelope proteins (Envs) play a critical role in HIV infection. In a correct trimeric conformation, Env mediates virus–cell binding and fusion. Malfunctioning of this machinery renders virions incapable of infecting cells. Each HIV-1 virion carries 10–14 Envs, and therefore a defective Env may not necessarily render a HIV virion non-infectious, since other Env on the same virion may still be functional. Alternatively, it is possible that on a given virion either all the spikes are defective or all are functional. Here, we investigate Env conformations on *individual* virions using our new nanotechnology, “flow virometry”, and a panel of antibodies that discriminate between various Env conformations. We found that the majority of HIV-1 virions carry either only trimeric (“functional”) or only defective spikes. The relatively small subfraction of virions that carry both functional and nonfunctional Envs contributes little to HIV infection of human lymphoid tissue *ex vivo*. The observation that the majority of virions exclusively express either functional or nonfunctional forms of Env has important implications for understanding the role of neutralizing and non-neutralizing antibodies in the immune control of HIV infection as well as for the development of effective prophylactic strategies.

## Introduction

HIV is one of the most variable viruses because of its high mutation rate and the conformational plasticity of *env* ^1^. The rapid evolution of this virus within an infected individual allows it to evade host cellular and antiviral humoral immune responses, the latter driving evolution of the viral envelope glycoprotein^2^. Initial antibody responses are to non-neutralizing epitopes and although most subjects go on to develop autologous neutralizing responses over a period of weeks to months, the virus rapidly escapes them rendering such responses obsolete. Some 10–25% of infected individuals go on to develop neutralizing antibodies of some breadth, although these also fail to inhibit replication of their autologous virus^3–5^.

The HIV envelope plays a critical role in HIV infection. Functional HIV envelope proteins (Envs) or spikes are trimers consisting of three homodimers of gp120 and trans-membrane gp41 subunits linked to each other via non-covalent bonds^6^. The correct conformation of Env is critical for the virus to bind to cell receptors (CD4) and coreceptors (CCR5/CXCR4) and to undergo the complex conformational change required for plasma membrane fusion and viral entry. Malfunctioning of this machinery renders virions incapable of binding/fusion with cells. This malfunctioning may be mediated by incorporation of dysfunctional forms of Env into the virion membrane or by the instability of incorporated functional Env. Indeed, a vast majority of virions within any viral population are thought to be non-infectious^7^. Several ways by which the HIV Env can be dysfunctional have been described. These include uncleaved Env, dimeric or monomeric Env, and gp41 stumps^8–10^. Each HIV-1 virion carries 10–14 spikes^11^. In principle, it is possible that on a given virion all the spikes are either defective or all are functional, rendering the former virion defective and the latter virion infectious. Alternatively, virions may carry both functional and non-functional Envs in different conformations. Determining which of these possibilities actually exists is important both for understanding of the basic mechanisms of HIV infection and for the development of new therapeutic and prevention strategies, in particular vaccines.

Earlier, we^12^ investigated the distribution of various functional and non-functional forms of Env on HIV preparation using immunoadsorbtion. This bulk analysis revealed the existence of subpopulations of virions carrying distinct forms of Env. Here, we further study this problem by investigating the conformation of Envs on *individual* virions using our new technology of flow virometry and a panel of antibodies that discriminate between various gp120 conformations^13^.

We found that the majority of virions do not carry defective and trimeric spikes simultaneously. Accordingly, the depletion of the virions that carry defective Env only mildly decreases the infection of human lymphoid tissue.

## Results

To analyze native HIV virions individually we (i) coupled magnetic nanoparticles (MNPs) with anti-HIV antibodies, (ii) stained MNPs with fluorescent anti-human IgG1 Fab fragments, (iii) captured HIV virions with antibody-coupled MNPs, (iv) stained the resultant complexes with fluorescent “detection” antibodies against antigens of interest, (v) separated HIV-MNP-detection antibody complexes from free antibodies and non-captured virions in a strong magnetic field using magnetic columns, and (vi) analyzed complexes with a flow cytometer set to be triggered by fluorescence, rather than by light scattering^14^ (Fig. S1).

We used laboratory strains of HIV-1 and a transmitted/founder virus. First, we used a fluorescently labeled HIV-1 preparation^15^ to test whether we captured the majority of the virions that are recognized by the capture antibodies. We captured fluorescently labeled HIV-1_BaL_ via MNPs coupled to 2G12, which recognize clusters of high mannose type glycans on the outer domain of gp120. After incubation of 2G12-MNPs with the viral preparation we isolated the resulting complexes on magnetic columns. The fraction that was retained on the column was eluted and analyzed by flow cytometry as described in the Methods. We incubated those virions that were not captured on the first run again with 2G12-MNPs, isolated the re-captured virions on a magnetic column, eluted this fraction and analyzed by flow cytometry. The results of this experiment are presented in Fig. S2. On average, on the first run we captured ~95% of virions recognized by 2G12 since in the flow-through fraction we recaptured 5 ± 2.8% (n = 3) of virions recognized by this antibody. This result is in agreement with the earlier evaluated efficiency of our virion capture technique^14^.

**Figure 1:**
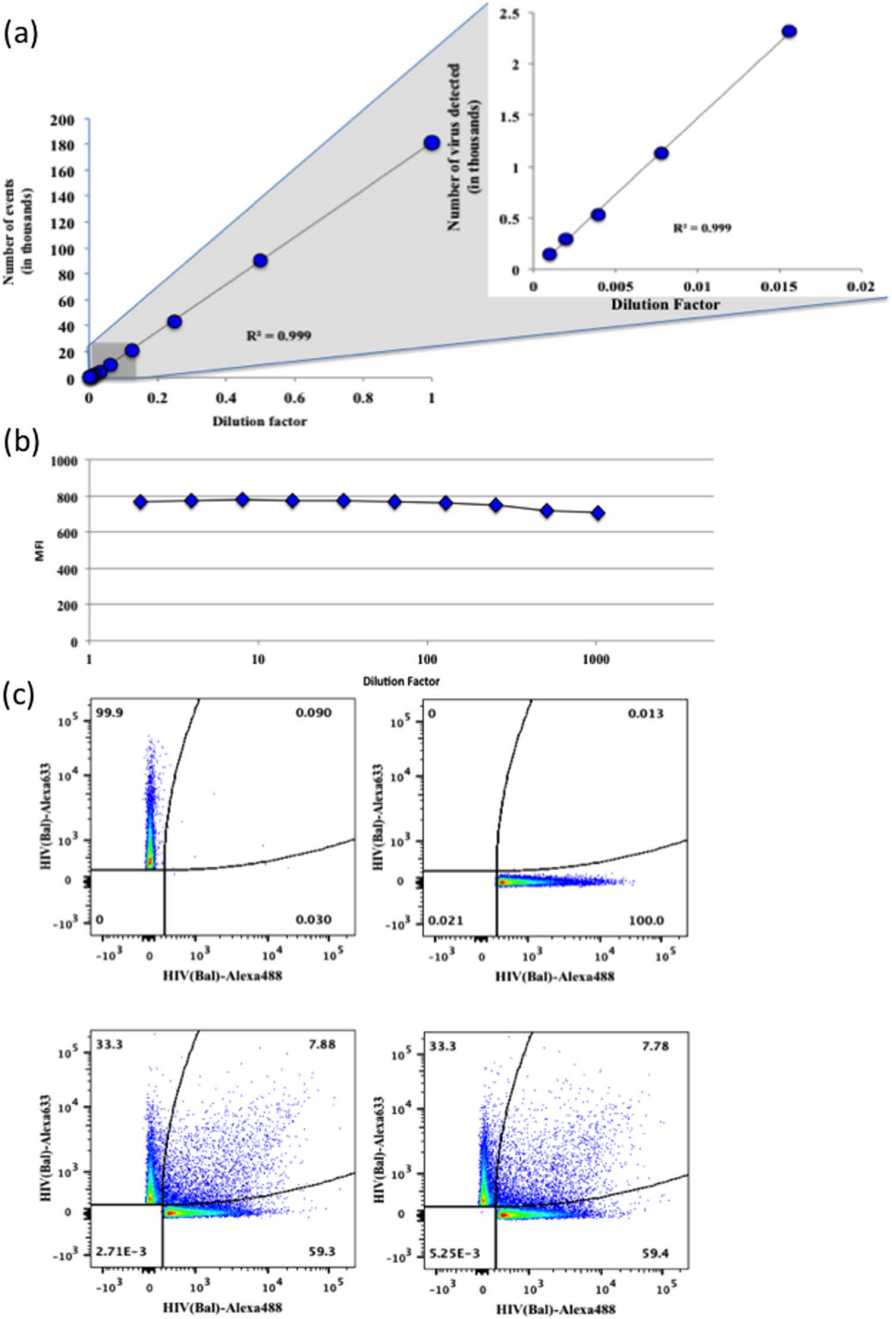
Analysis of individual HIV virions. HIV_BaL_ labeled with Alexa Fluor 488 5C Maleimide was captured with 2G12-MNPs, isolated on magnetic column and serially diluted from 2 to 2^10^ fold. The events were acquired in a fixed volume of 80 μl in duplicate using a High Throughput Sampler (HTS) at a flow rate of 0.5 μl per second on the LSRII flow cytometer, set to be triggered by Alexa Fluor 488 fluorescence. (**a**) Presented are numbers of events as a function of the dilution factor. Right panel is blow up of points near 0. (**b**) Median fluorescence intensity (MFI) of two-fold serially diluted events. (**c**) HIV_BaL_ preparation was split into two fractions; one fraction was labeled with Alexa Fluor 633 5C Maleimide (upper left panel) the other fraction was labeled with Alexa Fluor 488 5C Maleimide (upper right panel). The two differently labeled HIV fractions were mixed (lower left panel) and all the fractions were captured with 2G12-MNPs and isolated on magnetic columns. Alternatively, mixed fraction after capture with 2G12-MNPs was incubated with detection antibodies (lower right panel). The resultant complexes were eluted from the columns and analyzed on flow cytometer triggered on fluorescence.

Next, we verified that under our protocol we visualize predominantly individual virions. Particle sizing demonstrated that ~90% of the captured virions or of captured virions treated with detection antibodies, roughly correspond to the size of individual viral particles (+MNPs)^16^ (Fig. S3b,d). Filtering of the preparation through a 0.22 µm filter prior to capture did not significantly change the size distribution of the population (Fig. S3c,e). Next, using a fluorescent-labeled HIV preparation, we demonstrated a strong linear correlation between the number of events and dilutions over the range of 2^10^ fold (Fig. 1a), whereas the MFI of the population did not change over same dilution range (Fig. 1b). Nevertheless, if the aggregates were stable, their numbers upon dilution would still follow a linear relation with the dilution. To exclude this possibility we divided viral preparation into two fractions and labeled one fraction with Alexa Fluor 488 C5 Maleimide and the second fraction with Alexa Fluor 633 C5 Maleimide^14^. After mixing these two differently stained viral suspensions we captured virions with MNPs on a magnetic column analyzed them with a flow cytometer. Only 8.1 ± 0.2% of the events were double colored aggregates (Fig. 1c). Addition of PG9 detection antibodies to the captured virions did not change the fraction of dual-colored events (7.7 ± 0.1%).

Having verified the high efficiency of the capture and that the vast majority (>90%) of the events represent individual virions we moved to the investigation of the distribution of envelope conformations on these individual viral particles. Initially, we evaluated whether HIV-1_BaL_ virions can simultaneously carry trimeric and defective Envs as revealed by the binding of antibodies that are considered to be specific for functional or defective Env spikes. To answer this question we used a panel of monoclonal antibodies^17–24^ (Table S1) to distinguish different envelope conformations. We checked (Table S2) that these antibodies did not compete with each other, thus their differential binding reflects the presence of specific conformation of Env rather than antibody interference. As a positive control for the competition assay we used PG9 and PGT145 that compete for binding to very closely-situated epitopes on Env in trimeric conformation. This pair was not used in combination as detection antibodies in the experiments below.

**Figure 2:**
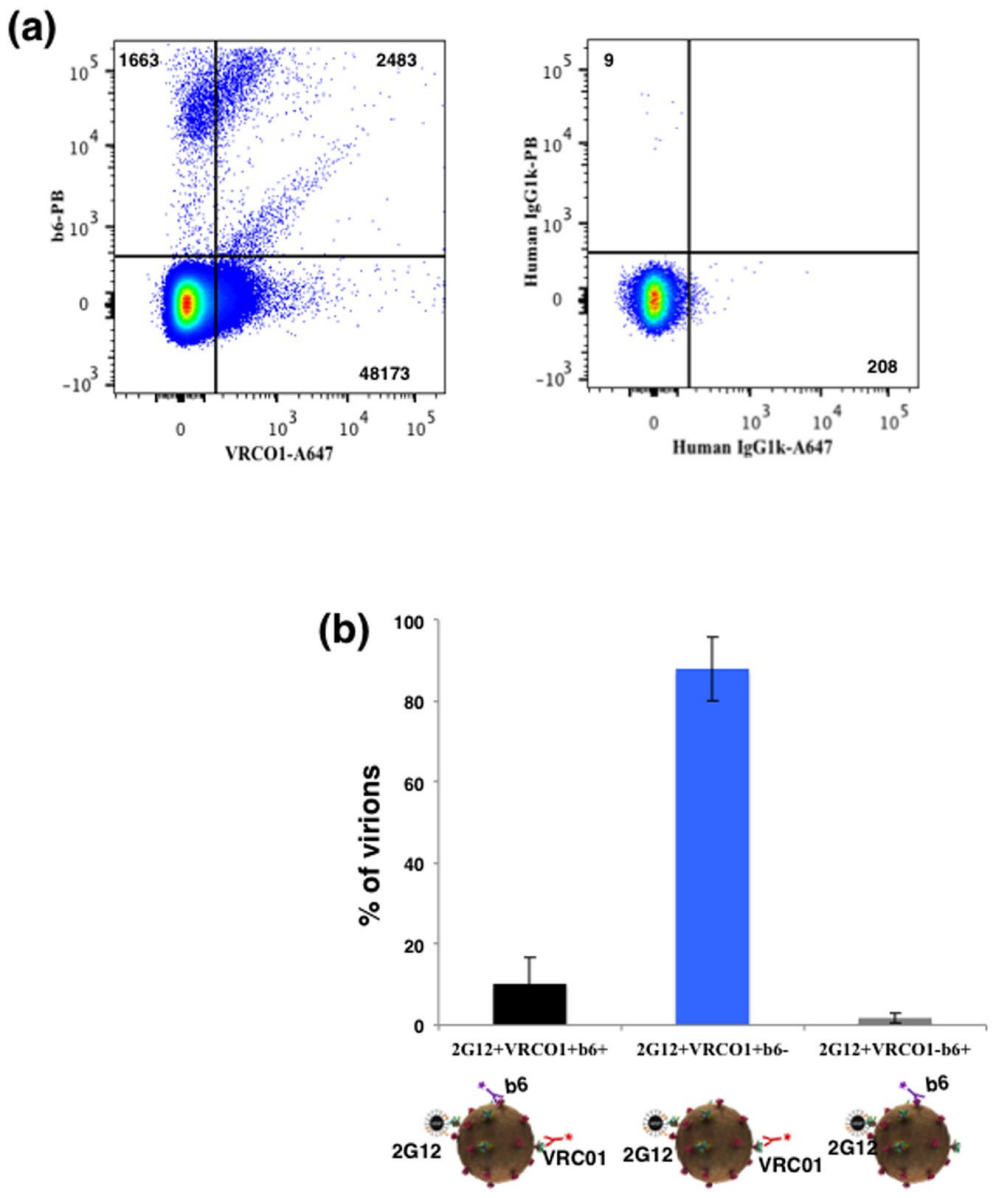
Flow virometry of HIV-1_BaL_ virions captured with 2G12-MNPs. (**a**) Virions were captured with 2G12-MNPs and stained with: AlexaFluor 647-labeled VRC01, which predominantly recognizes trimeric Envs and Pacific Blue-labeled b6, which recognizes uncleaved and non-trimeric Envs (left panel) or with isotype control antibodies (right panel). Indicated are the numbers of events. A representative experiment out of three is shown. (**b**) Distribution of 2G12-captured virions positive for VRC01 (recognizing predominantly trimeric Envs) and/or for b6 (recognizing predominantly uncleaved and non trimeric forms of Envs). Each bar represents mean ± SEM of three experiments.

We captured HIV-1_BaL_ virions with 2G12-MNPs since it recognizes different Env forms^12^ and revealed them with two antibodies (Fig. 2a), VRC01, which preferentially recognizes the CD4 binding site (CD4bs) on all gp120 forms and b6, which preferentially recognizes the CD4bs on uncleaved gp160 as well as non-trimeric forms of gp120^19, 25^. On average 88 ± 7.9% (n = 3) of 2G12-captured virions were positive for VRCO1 and negative for b6. Of the rest, 10.2 ± 6.7% (n = 3) were dual positive for VRC01 and b6, while 1.8 ± 1.2% were positive for b6 only (Fig. 2b).

Thus, if a virion carries at least one Env recognized by VRC01, there is a low probability that there are other Envs on the same virion recognized by b6. To further evaluate the probability of individual virions carrying trimeric and defective envelopes we captured virions again with 2G12-MNPs but probed envelope display using a more trimer-specific antibody, PGT145. We observed a pattern similar to that with VRC01 where the majority of viruses were either positive for b6 or PGT145, however in this case a larger fraction of captured virions were b6+ only than PGT145+ only. Nevertheless again virions positive for both forms of Env were in minority (Fig. S4).

**Figure 3:**
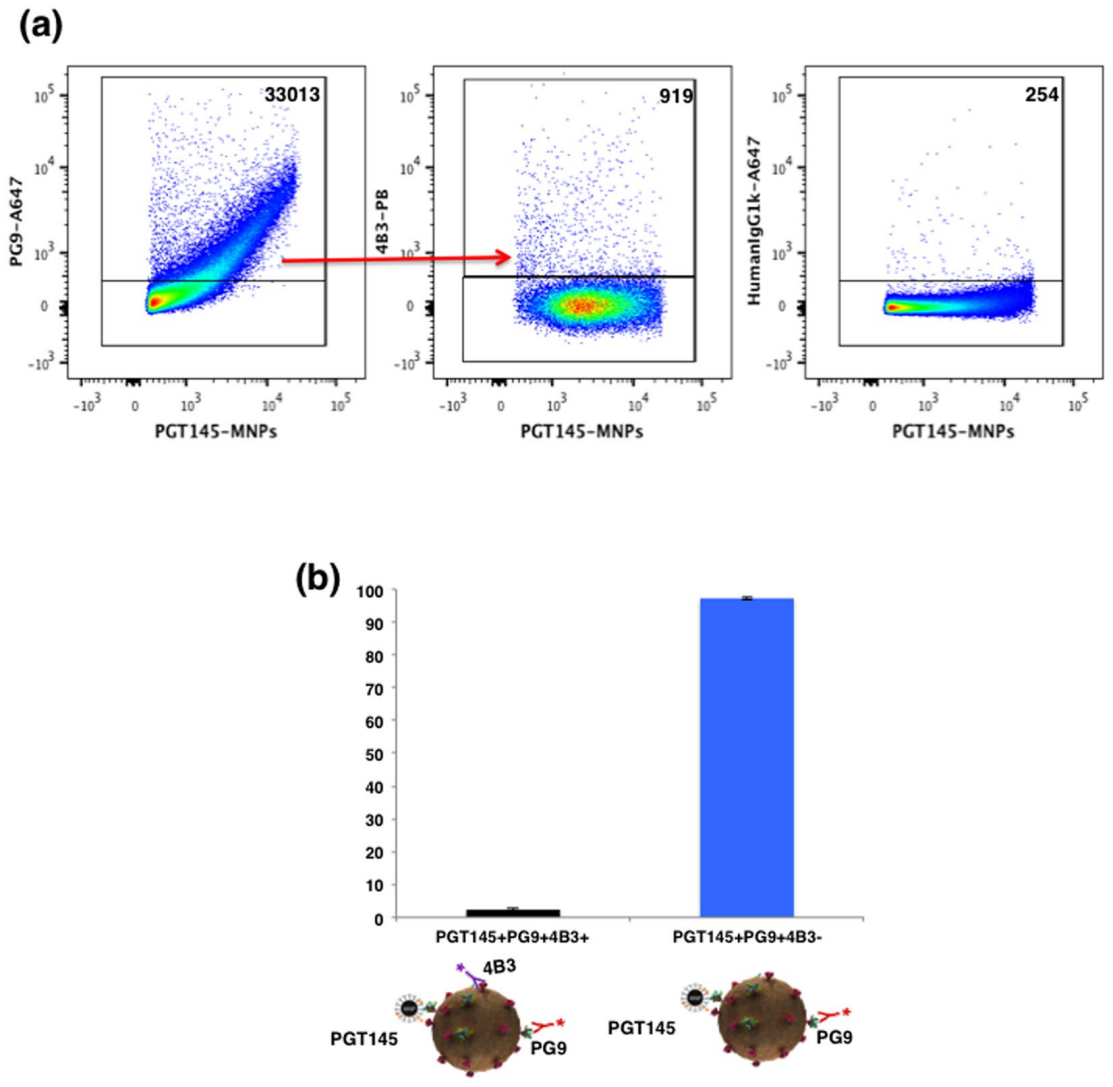
Flow virometry of HIV-1_BaL_ virions captured with PGT145-MNPs. (**a**) Virions were captured with PGT145-MNPs and stained with AlexaFluor 647-labeled PG9 (which recognizes trimeric Envs) and Pacific Blue-labeled 4B3 (which recognizes “stumps” or uncleaved Envs). Left panel: Virions were captured with PGT145-MNPs and stained with PG9 antibody. Central panel: PG9-positive PGT145-MNPs captured virions stained with 4B3. Right panel: isotype control staining. Indicated are the numbers of events. (**b**) Distribution of PGT145-captured virions positive for PG9 and 4B3. Each bar represents mean ± SEM of two experiments.

**Figure 4:**
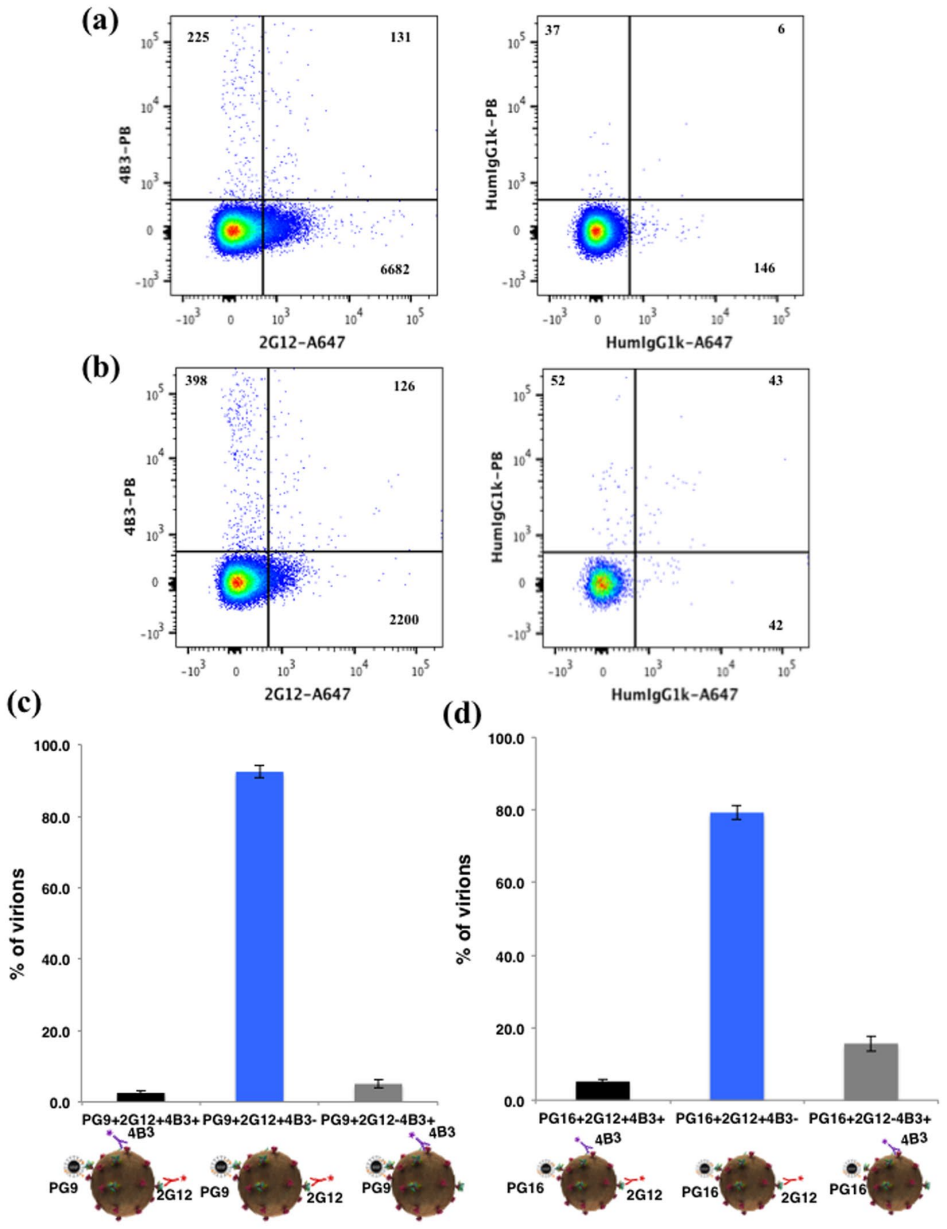
Flow virometry of HIV-1_BaL_ virions captured with PG9- or PG16-MNPs. (**a**) Virions were captured with PG9-MNPs and stained with two antibodies: AlexaFluor 647-labeled 2G12 and Pacific Blue-labeled 4B3 (left panel) or with isotype control antibodies (right panel). A representative experiment out of three is shown. (**b**) Virions were captured with PG16-MNPs and stained with two antibodies: AlexaFluor 647-labeled 2G12 and Pacific Blue-labeled 4B3 (left panel) or with isotype control antibodies (right panel). A representative experiment out of three is shown. (**c**,**d**) Distribution of PG9-captured (**c**) and PG16-captured (**d**) virions recognized by 2G12 and/or by 4B3. Each bar represents mean ± SEM of three experiments.

In the above–described experiments we used b6 as an antibody that reveals a particular defect in Env, namely uncleaved gp160. In the next series of experiments, we used an antibody that recognizes another Env defect, 4B3 that binds to gp41 revealing “stumps” devoid of gp120. We captured virions with PGT145-MNPs and detected stumps with 4B3. Also, we probed virions with PG9, an antibody that preferentially recognizes trimeric Env. Figure 3 demonstrates that in this population of virions that carry trimeric Env, a minority of virions also carry defective Envs that are recognized by 4B3. Again, we changed capture and detection antibodies and captured virions with PG9, while staining with 4B3 and 2G12. As presented in Fig. 4(a,c) with this combination of antibodies the results remain the same: the majority of the analyzed virions that were captured through their trimeric Env do not carry a defective Env recognized by 4B3 while only few percent were positive for all the antibodies. A similar result was obtained in the experiments where we captured virions with PG16 (Fig. 4b,d), another antibody that recognizes trimeric Env. Similar to the previous experiments the vast majority of virions carried trimeric Envs only.

Thus, the results with PG9- and PG16-captured virions are in agreement with our initial experiments with 2G12-MNPs, which indicated that if a virion has at least one trimeric Env, there is a low probability of the presence on the same virion of Envs recognized by 4B3 antibodies.

**Figure 5:**
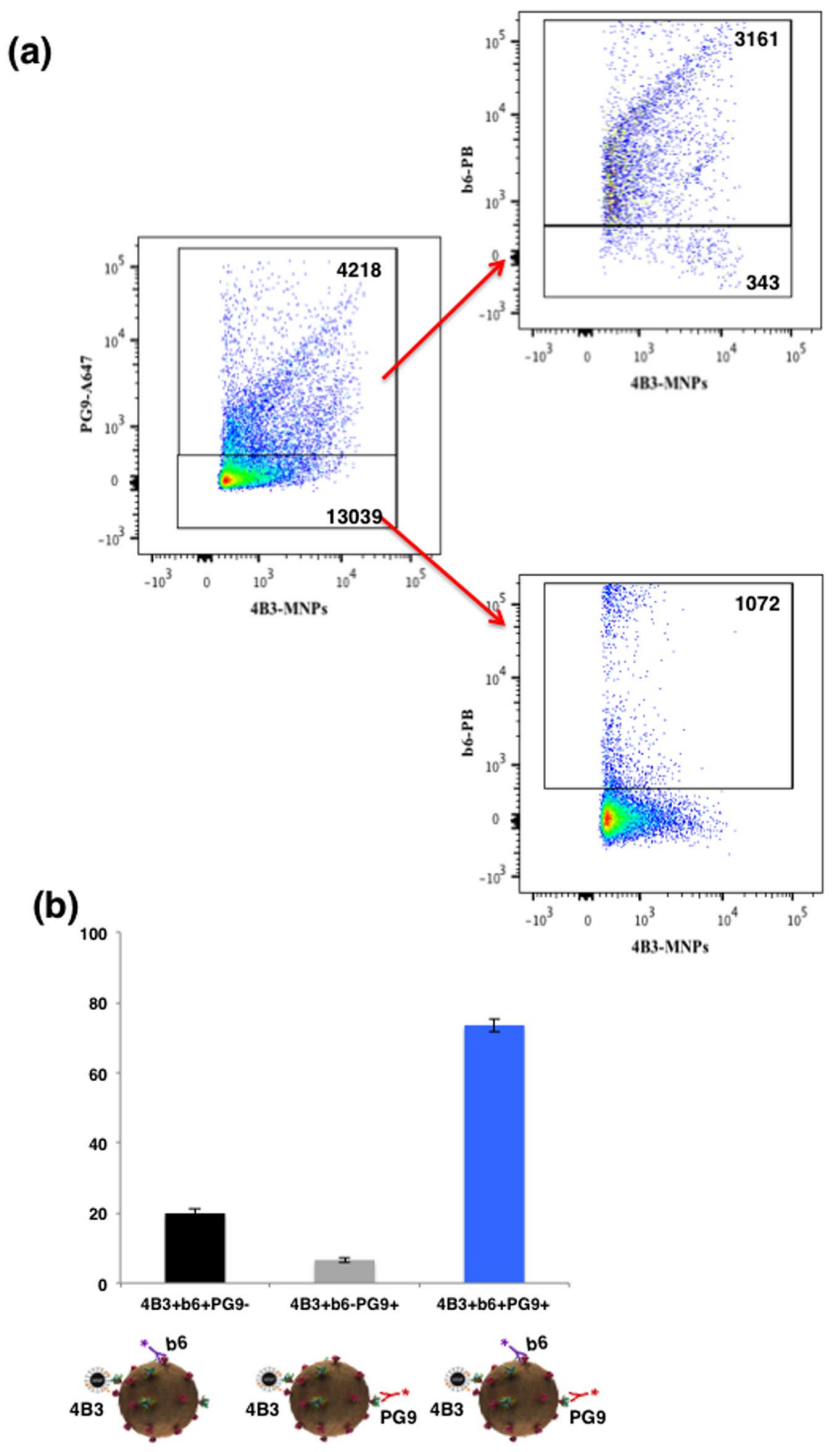
Flow virometry of HIV-1_BaL_ virions captured with 4B3-MNPs. (**a**) Virions were captured with 4B3-MNPs and stained with two antibodies: AlexaFluor 647-labeled PG9 antibodies and Pacific Blue-labeled b6. Left panel: Virions were captured with 4B3-MNPs and stained with PG9; upper right panel: 4B3-MNP-captured virions positive for PG9 and stained with b6; lower right panel: 4B3-MNP-captured virions negative for PG9 and stained with b6. Indicated are the numbers of events. A representative experiment out of three is shown. (**b**) Distribution of 4B3-captured virions positive for PG9 and b6. Each bar represents mean ± SEM of three experiments.

Finally, we captured virions with PGT145 and stained for both studied defects, uncleaved gp160 and “stumps”, with a mixture of 4B3 and b6 antibodies and found that in this fraction of virions the two defects largely segregate with only less than 10% of virions carrying both stumps and uncleaved gp160 recognized by these antibodies. However, these two types of defective Envs may coexist on the PG9 positive virions in the experiments where we selectively captured such virions with 4B3-MNPs (Fig. 5).

To investigate whether the relative lack of Env mosaic virions demonstrated above for HIV_BaL_ is true for another HIV-1 isolate, we extended the experiments reported above to the isolate SF162. We captured HIV-1_SF162_ with PG16-MNPs and stained them with 4B3 antibodies, in experiments similar to those described above for BaL. The results of these experiments also show that for HIV-1_SF162_ both defective and trimeric Envs co-exist on a minor fraction of virions. However, for this strain of HIV-1 this fraction was larger than a corresponding fraction of HIV-1_BaL_: 66.6 ± 3.3% of the SF162 virions captured through trimeric spikes do not carry Env forms that could be recognized by 4B3 antibodies. 33.3 ± 3.3% (n = 3) of virions were mosaic for PG16 and 4B3 expressing a combination of trimeric and “stump” Envs.

**Figure 6:**
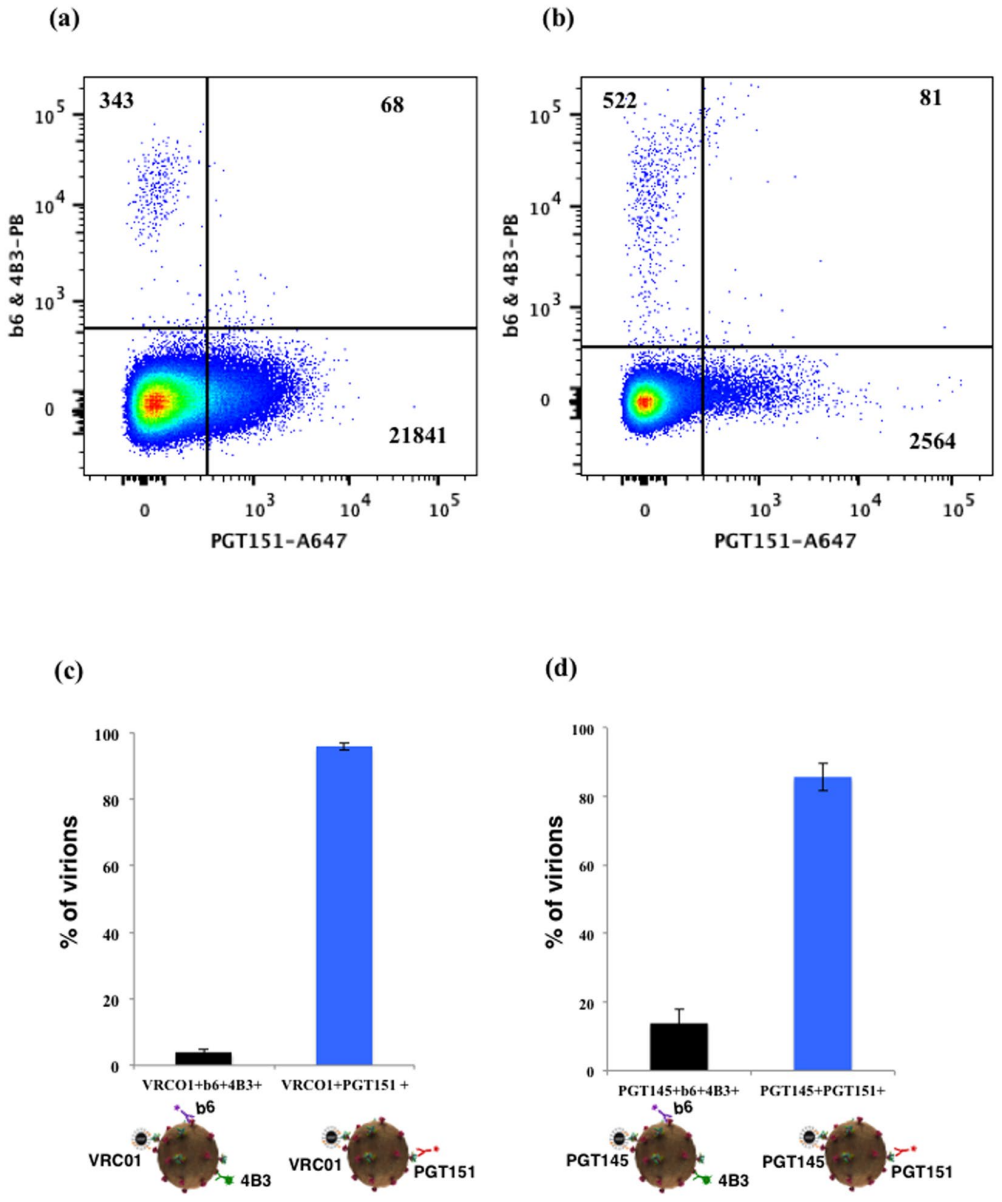
Flow virometry of T/F HIV-1_CH162_ virions. (**a**,**c**) Distribution of VRC01-MNPs–captured virions stained with three antibodies: AlexaFluor 647-labeled PGT151, Pacific Blue-labeled b6 and 4B3. Each bar represents mean ± SEM of three experiments. (**b**,**d**) Distribution of PGT145-MNPs–captured virions stained with three antibodies: AlexaFluor 647-labeled PGT151, Pacific Blue-labeled b6 and 4B3. Each bar represents mean ± SEM of three experiments.

The above-analyzed viruses were of laboratory strains. Below we applied our analysis to a transmitted/founder (T/F) isolate CH162. We captured this virus with MNPs coupled to either VRC01 or PGT145 and stained with PGT151 and a mixture of b6 and 4B3. The defective Envs were present on less than 8% of virions captured by VRC01 or PGT145, while more than 90% of the captured viral particles were positive for PGT151 only (Fig. 6a,b). A similar distribution pattern was observed when we used PGT145-MNPs to capture virions and stained them with PGT151 and a mixture of b6 and 4B3. Again about 90% of CH162 virions captured by PGT151 did not bind either b6 or 4B3 antibodies (Fig. 6c,d).

Thus, in the case of both VRC01-MNPs and PGT145-MNPs capture, the vast majority of virions were negative for b6 and 4B3 antibodies binding.

In all the above-described experiments we identified HIV-1 virions by capturing them with MNPs through binding to one surface antigen and detecting positive virions with labeled antibodies that recognize another antigen. However, some of the captured virions may not carry antigens that are recognized by any combination of detection antibodies that were used. To include these virions in our analysis we used a preparation of HIV-1_BaL_ fluorescently labeled with Alexa Fluor 488 through their SH groups^15^. The procedure of fluorescence labeling did not change the antigenic properties of virions as the results of flow analysis of both fluorescently labeled and non-labeled preparations captured with VRC01-MNPs and stained with 2G12 were similar (Fig. S5).

**Figure 7:**
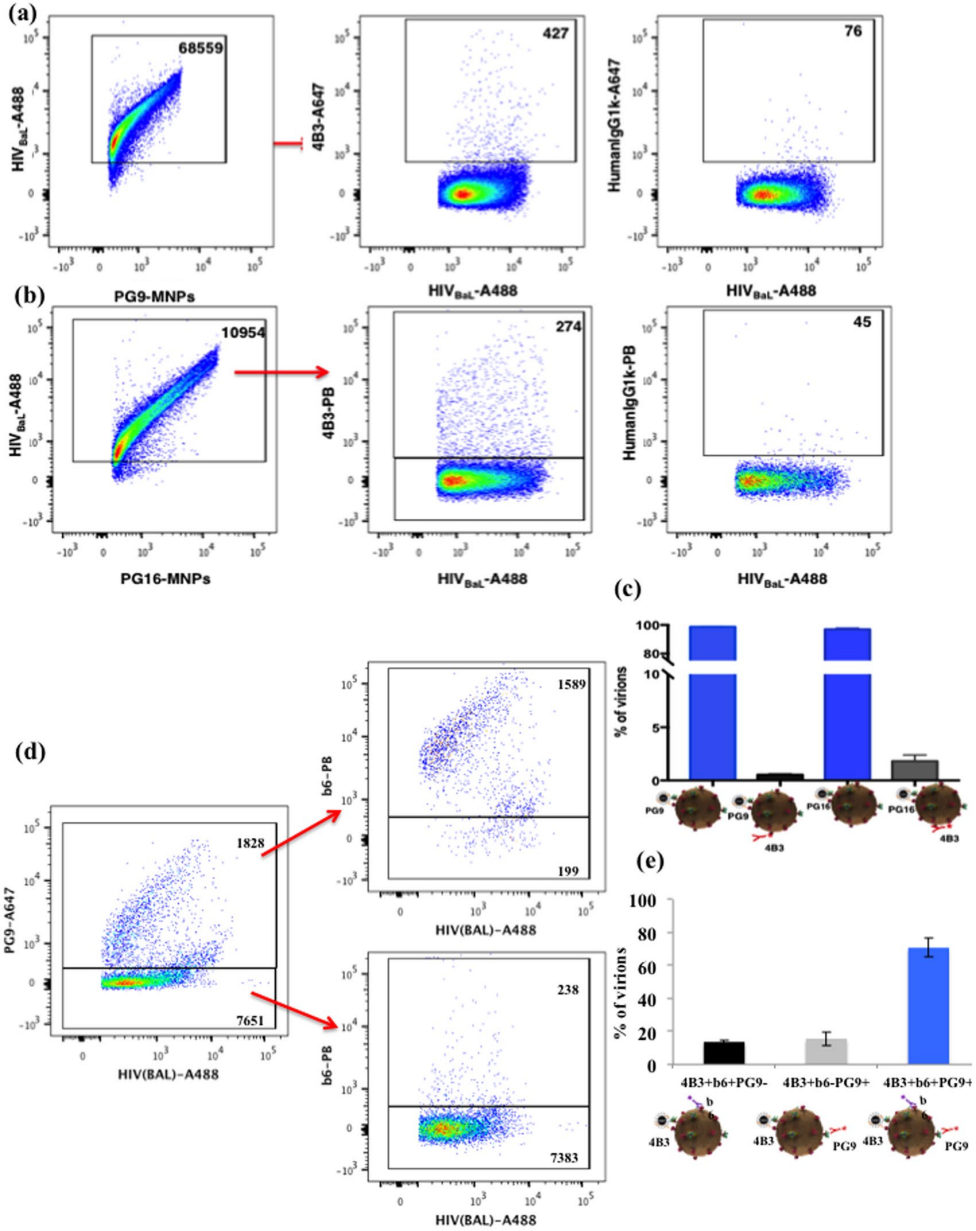
Flow virometry of fluorescence-labeled HIV-1_BaL_ virions. (**a**) Alexa Fluor 488 -labeled virions were captured with PG9-MNPs (left panel) and stained with AlexaFluor 647 4B3 antibodies for the presence of stumps/uncleaved Envs (center panel) or with AlexaFluor 647-labeled isotype control antibodies (right panel). Indicated are the numbers of events. A representative experiment out of three is shown. (**b**) Alexa Fluor 488 -labeled virions were captured with PG16-MNPs (left panel) and stained with Pacific Blue-labeled 4B3 antibodies for the presence of stumps (center panel) or with Pacific Blue-labeled isotype control antibodies (right panel). Indicated are the numbers of events. A representative experiment out of three is shown. (**c**) Fractions of PG9-MNPs and PG16-MNPs captured Alexa Fluor 488 virions positive for 4B3. Each bar represents mean ± SEM of three experiments. (**d**) Alexa Fluor 488 -labeled virions were captured with 4B3-MNPs and stained with two antibodies: AlexaFluor 647-labeled PG9 and Pacific Blue-labeled b6. Left panel: Virions were captured with 4B3-MNPs and stained with PG9. Upper right panel: 4B3-MNP-captured virions positive for PG9 and stained with b6. Lower right panel: 4B3-MNP-captured virions negative for PG9 and stained with b6. Indicated are the numbers of events. A representative experiment out of three is shown. (**e**) Distribution of 4B3-MNPs–captured Alexa Fluor 488 labeled virions stained with two antibodies: AlexaFluor 647-labeled PG9 and Pacific Blue-labeled 4B3. Each bar represents mean ± SEM of three experiments.

We used fluorescently labeled HIV-1_BaL_ to evaluate the heterogeneity of Env on individual virions that may have been missed when we analyzed non-fluorescent virions whose detection requires binding of at least one anti-Env antibody. First, we captured virions that carry trimeric Envs using either PG9-MNPs or PG16-MNPs and stained captured virions for stumps with 4B3 antibodies. Virions carrying trimeric spikes were mostly negative for the presence of stumps (Fig. 7a,b). On average only 0.5 ± 0.1% (n = 3) of the PG9-captured and 1.8 ± 0.3% (n = 3) of the PG16-captured virions also bound 4B3.

Next, we captured fluorescent virus with 4B3-MNPs. In agreement with our results using non-fluorescent virus, this fraction contains virions that are highly mosaic including virions that are positive for b6 and PG9. These latter virions constituted 73.5 ± 1.9% (n = 3) of all the virions that are recognized by the detection antibodies. Importantly, using intrinsic fluorescence we are no longer restricted to virions that are recognized by detection antibodies but could also visualize 4B3-MNP-captured virions negative for both b6 and PG9. The latter virions constituted the majority, 80.7% of 4B3-captured virions (Fig. 7c).

**Figure 8:**
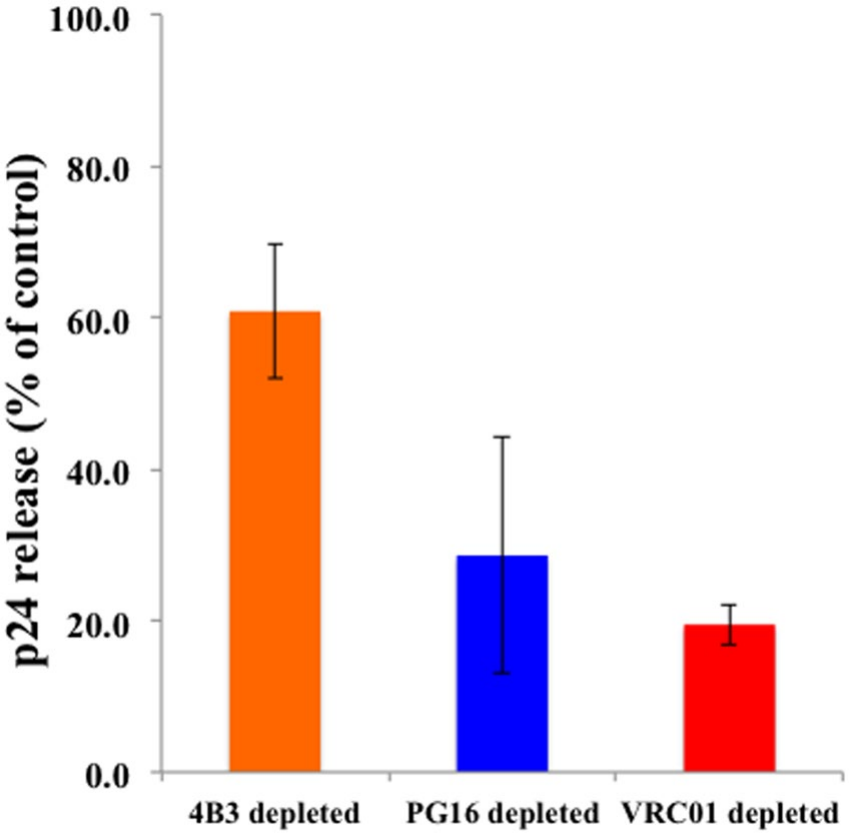
Infection of human lymphoid tissue *ex vivo* with MNP-depleted HIV preparations. Virions were captured with either 4B3-MNPs, PG16-MNPs, VRC01-MNPs or with isotype control (mouse IgG) MNPs. Captured virions were retained on magnetic columns, and the “flow-through” fractions, depleted of the captured virions, were used as inoculums. Infection of the tissues was evaluated from the amount of p24 released to the medium between day 6 and day 12 post-inoculation. Infection of the control flow-through fraction was considered to be 100%. Bars represent mean ± SEM of six experiments or five experiments. (In one tissue PG16-MNPs did not reduce infectivity and this experiment was excluded).

Thus, flow virometry with different sets of antibodies that recognize Envs in different conformation revealed that the majority of virions carry either only trimeric or only defective Envs and only the minority carries both. To check whether this fraction plays a significant role in infection we performed experiments to evaluate the infectivity of viral preparations. We infected blocks of human lymphoid tissues^26^ either with viral preparations from which virions with defective spikes were removed with 4B3-MNPs or with preparations from which virions carrying trimeric spikes recognized by PG16-MNPs or VRC01-MNPs were removed. To prepare these viral preparations, we incubated viruses with one of these MNPs and passed them through magnetic columns. For infection, we used the “flow-through” fractions depleted of the appropriate virions. As a control we used the “flow through” fraction of virions incubated with MNPs coupled to mouse IgG (isotype control). 4B3-MNPs depleted viral preparation infected human lymphoid tissues ~40% less (60.8 ± 15.5%, n = 6) than the control preparation. In contrast, viral preparations depleted with PG16-MNPs or VRC01-MNPs were significantly less infectious (p = 0.01 and p = 0.03, respectively). These preparations infected human lymphoid tissue to the level of 28.6 ± 8.8% (n = 6) and 19.5% ± 2.7 (n = 5) of control, respectively (Fig. 8).

## Discussion

To bind and fuse with a target cell membrane, HIV-1 employs *env*-encoded gp160 that is cleaved into gp41 and gp120 homodimers and forms a functional trimeric spike on the viral surface. During the course of binding and fusion with the plasma membrane this complex has to undergo dramatic conformational changes, now well described^6, 27–29^. Like all efficient machines it is functional in only one state but may be dysfunctional in many others. Several defects of spikes have been described in the literature. In particular, gp160 may not be cleaved into gp41 and gp120, spikes can be formed by dimers or monomers rather than by trimers, the gp120 component of the spike can be lost from the virus leaving only a gp41 “stump” on the viral surface^8, 10^.

However, even a defective spike may not necessarily render a HIV virion non-infectious since other spikes on the same virion may still be functional. Each viral particle contains approximately 14 spikes^11^, which in principle may function independently. How many functional spikes are sufficient to make a virion infectious is a matter of debate but is thought to require 1 to 3 functional spikes^30–32^. If indeed only a few spikes need to be functional for the virus to be infectious it is possible that HIV-1 virions are predominantly mosaic in terms of exhibiting both functional and non-functional Envs.

Our early bulk analysis^12^ already demonstrated that distinct forms of Env may be present in the same viral preparation. Here, we were able to go further and to identify different forms of Env on *single* HIV particles and to analyze the distribution of different forms of Envs on *single* HIV-1 virions using our newly developed technique, “flow virometry”^14^.

Individual HIV virions were captured with 15-nm magnetic nanoparticles (MNPs) decorated with (“capture”) anti-Env or anti-gp41 antibodies that revealed so called stumps, and stained with fluorescent (“detection”) antibodies against other Envs’ or stump epitopes. MNPs were also made to fluoresce by decorating them with fluorescent Fab fragments that bind to the anti-gp120 (Zenon). The MNP-antibody-virion complexes were separated from non-bound fluorescent antibodies and their aggregates in a magnetic field, thus isolating captured virions (although together with “empty” MNPs, which to avoid viral aggregation are in excess). Thus, although the virus capture is highly efficient, the majority of MNPs have no virus to capture. This is similar to the routine cell immunostaining when fluorescent antibodies are added in excess. MNPs that did not capture virions can be easily ignored because when the resultant suspension is analyzed in a flow cytometer, each virion is defined as an event positive both for MNP (stained with a Zenon) and for at least one more fluorophore that is associated with a second (detection) antibody thus ignoring “empty” MNPs. Here, we applied “flow virometry” to evaluate heterogeneity of Env forms on individual HIV-1 virions.

Earlier published experiments indicated that with a protocol similar to one used in the current work, more than 90% of the flow events represent individual viral particles^14^. We confirm and extend these finding using particle sizing, filtering the viral preparations to remove aggregates, determining correlation between the number of events and dilutions over the range of 2^10^ fold, and evaluating aggregates by capturing virions from a mixture of Alexa Fluor 633- and Alexa Fluor 488-labeled virions. These results together with earlier published evidence^14, 33, 34^ indicate that our approach predominantly visualizes individual virions stained with fluorescent antibodies.

The technique of flow virometry focuses on virions that carry a particular antigen of interest detected by specific antibodies. In the current work we used a panel of antibodies that distinguish Envs in different conformations (Table S1). Potentially gp120 shedding may interfere with our analyses, however we believe that the potential for shedding in our experiments is minimal given the incubation period for antibody-virion interaction is limited to 1 hr. Previous studies have demonstrated that significant antibody induced shedding of gp120 is inefficient, taking more than 10 hours, in contrast to CD4-IgG that induces rapid shedding following dramatic conformational change^35^.

First, we evaluated the efficiency of the MNP capture. Towards this goal we used 2G12 antibodies to capture fluorescently labeled HIV-1_BaL_. Following capture of virus by 2G12-MNPs, we collected the flow-through fraction from the magnetic column and again incubated it with 2G12-MNPs to capture virions that may have been missed in the first run. In agreement with earlier data^14^ we found that less than 5% of virions of interest may have remained uncaptured in the first run. Thus, due to the high efficiency of capture we are able to capture for analysis virtually all virions that carry antigens of interest.

Now it became possible to address what combinations of Env types are present on the surface of HIV virions. For this we combined different capture antibodies (coupled to MNPs) with different detection antibodies. Here detection antibodies can accurately reveal distribution of distinct specific antigens on the surface of individual virions provided that they do not interfere with their respective binding. To exclude the latter possibility we evaluated potential interference for each pair of detection antibodies used in the current work. Towards this goal we captured virions with MNPs coupled to 2G12 that has a broad recognition pattern and compared the number of positive virions when stained with only one detection antibody with the number of positive virions stained with the same antibody but in combination with the second antibody. For none of the pairs, when used as detection antibodies, were these numbers significantly different indicating the lack of competition for binding to virions.

The results obtained from staining of individual virions with mixtures of antibodies that recognize trimeric and “defective” conformations of Env indicate that only a minor fraction of virions carry both trimeric and defective Envs (uncleaved gp160 recognized by b6 antibodies or “stumps” recognized by 4B3 antibodies). The majority of virions carry either defective or trimeric Envs. Indeed, all tested combinations of capture and detection antibodies consistently revealed only a minority of virions that simultaneously carry defective forms of Envs and other forms of Envs recognized by antibodies that bind either predominantly or exclusively to trimeric Envs.

In a deeper analysis we investigated this minor fraction of “mosaic” virions for simultaneous presence of different defective Env forms, recognized by b6 and 4B3. We found such virions but they constitute “a minority of a minority”: in virions captured with PGT145-MNPs, the two defects largely segregate to different virions.

The experiments discussed above were performed with a prototypic CCR5-tropic HIV isolate BaL. To investigate whether a similar distribution of defective and trimeric Env occurs with other isolates we analyzed another laboratory strain of HIV-1, SF162. We found that on this isolate trimeric and defective Envs also largely segregate to different virions, however the extent of this segregation was less than on BaL virions and approximately one third of virions carry both trimeric Envs (recognized by PG16) and “stumps” (recognized by 4B3 antibodies).

The lack of prevalence of the virions on which trimeric and defective Envs co-exist was even more typical for the T/F variant CH162. Analysis of CH162 virions captured either with PGT145-MNPs or VRC01-MNPs and stained with PGT151 and a mixture of 4B3 and b6 antibodies revealed that the majority of virions carry neither forms of defective Envs and more than 90% of virions carry trimers only.

Further systematic application of flow virometry to a larger panel of different isolates may show whether the size of the fraction of virions, which carry several forms of Envs is related to biological properties of the viruses. In particular, it should be investigated whether various T/F viruses have a different distribution of Envs compared to laboratory strains or chronic isolates.

Here, we evaluated one of these functions, namely the relative ability to infect human lymphoid tissue *ex vivo*. In this system lymphoid tissue preserves its cytoarchitecture, supports HIV infection without artificial stimulation or activation and faithfully reflects certain important aspects of lymphoid tissue *in vivo* ^26^ where critical events of HIV infection occur. We evaluated whether the depletion of virions that carry “stumps” significantly decreases infectivity of the preparation. This would be the case if, in contrast to our flow virometry data, a significant fraction of trimeric functional Envs are present on virions that also carry stumps. We depleted viral preparation of virions that carry Env recognized by 4B3 and inoculated *ex vivo* human lymphoid tissue with this preparation.

The results of these experiments demonstrate that the majority of virions that are recognized by 4B3 are functionally defective. In contrast, capture of virions expressing functional Env with PG16-MNPs resulted in a substantial loss of infectivity and therefore depletion of infectious virions from the total population. The remaining infectivity of this preparation may be mediated by a few PG16-positive virions that have not been removed from the preparation, and that after many rounds of replication in the tissue result in detectable infection. Here, a fraction of infectious virions may not have been recognized by PG16 because of heterogeneity in glycosylation of Env as was reported earlier^36^. Depletion of virions expressing functional Env as recognized by VRC01 resulted in an even greater loss of infectivity reflecting the conserved nature of this epitope.

In conclusion, flow virometry demonstrated that the majority of HIV virions seem not to be mosaic but rather carry either only trimeric (“functional”) or only defective spikes as summarized by Venn diagrams (Fig. S6). However, this conclusion is subject to a number of potential caveats: First, our technology is only as precise as the specificities of the antibodies used. Although, the majority of the antibodies used are reported to preferentially bind to particular Env conformations (functional or non-functional) we cannot exclude additional low affinity for other Env forms and these affinities may be responsible for revealing some of the minor fractions observed. Second, since our technology can reveal at least three different antigens on a single virion that typically carries 13 or 14 spikes, at least three spikes of a particular conformation on the same virion can be seen, while a single spike may be below our level of detection. Third, for each capture MNP our analysis is restricted to the fraction of virions that carry Env recognized by the antibody that we chose for capture. These virions constitute fractions of various size^12^. However, our main conclusion as to partitioning of functional and defective Envs to discrete virion population was consistent across a range of antibody combinations and is also supported by the experiments with fluorescent virions in which we analyzed the total population of viral particles. Thus, even when taking into account the above-listed limitations, our results still indicate that the majority of virions in an HIV preparation display either only functional trimeric Envs or predominantly defective Envs. Only a relatively small sub-fraction of virions were mosaic with respect to carrying both functional and non-functional Envs. The contribution of this minor fraction to HIV infection of human tissue *ex vivo* seems to be small.

To our knowledge this is the first report on the quantitative analysis of distribution of Envs of different conformation on the surface of *individual* virions. The observation that the majority of virions exclusively express either functional or non-functional forms of Env has several important implications for understanding the role of neutralizing and non-neutralizing antibodies in viral acquisition and effective immune control. It is generally accepted that binding to functional trimers is a pre-requisite for neutralization, however the potential interaction of non-neutralizing antibodies with infectious virions is predicated on the assumption of mosaicism with respect to the expression of functional and non-functional forms on the same virion. The observed lack of mosaicism for the majority of infectious virions suggests that this all-or-nothing viral strategy likely aids immune evasion by subverting the focus of humoral responses to generate multiple non-neutralizing antibodies at no cost to infectious virions. Generation of such antibodies, which are predominant both in an HIV-infected patient and in vaccinated individual are highly unlikely to impede the initial events of HIV acquisition mediated by cell free virions that exclusively express functional forms of Env. By contrast, induction of antibodies that target functional Envs and thus target predominantly infectious viruses seem to be critical for the development of effective prophylactic strategies.

In general, determining the distribution of functional and non-function forms of HIV-1 Env at level of individual virions may prove critical both to our understanding of the basic mechanisms of HIV infection and for the development of new therapeutic and preventative strategies, in particular vaccines.

## Materials and Methods

### Viral preparation and labeling

For this study we used the following CCR5-tropic HIV viruses: BaL (HIV-1_BaL_) and SF162 (HIV-1_SF162_) (76 ng/ml and 37.8 ng/ml, respectively) that were produced in PBMCs by the VQA lab (Rush Medical School, Chicago, IL). Additional stocks of HIV-1_BaL_ and CH162 (accession number KC156126, infectious clone kindly provided by Dr. Christina Ochsenbauer) were obtained from chronically infected PM-1 T cells as previously described^37^. Stocks of HIV_BaL_ labeled with Alexa Fluor 488 C5 Maleimide (50 µM) or with Alexa Fluor 633 C5 Maleimide (38 µm) were kindly provided by Dr. Jeffrey Lifson. Additionally, stocks of Alexa Fluor 488 C5 Maleimide labeled BaL were prepared as follows. Prior to labeling, virions were concentrated by centrifugation in Amicon Ultra Centrifugation filter units (100 kDa, Merck Millipore), and sucrose purified by layering 900 µl of concentrated virus over 100 µl 20% sucrose, and centrifugation at 20,000× g for 1 hour at 4C. After centrifugation, the upper layer of supernatant was discarded and the sucrose fraction containing virion preparation was diluted in sterile PBS up to 1 ml. Virions were inactivated using N-Ethylmaleimide and simultaneously labeled with AlexaFluor 488 maleimide as described earlier^15^. Next, virions were depleted of CD45 using magnetic beads (Miltenyi Biotech) according to manufacturer protocol and pelleted at 150,000 g on 25% sucrose cushions for 60 minutes, and re-suspended in PBS. Concentrations of viral preparations obtained from PM-1 T cells after purification were assessed by p24 measurement (5.4 µg/ml and 263 ng/ml for non-labeled and AlexaFluor 488 maleimide-labeled BaL respectively).

### Coupling of monoclonal antibodies to magnetic nanoparticles

Human monoclonal antibodies (1 mg of PG9, PG16, 2G12, PGT145, or 4B3) recognizing different conformations of gp120 and gp41 on HIV-1 virions were coupled to 15 nm carboxyl terminated magnetic iron oxide nanoparticles (MNPs) (Ocean NanoTech, San Diego, CA) as previously described^14^. PG9, PG16, 2G12 and 4B3 were purchased from Polymun Scientific. PGT145 antibodies were produced by transfection of cloned expression vectors in 293-F cells (Invitrogen Carlsbad, California). The secreted human antibody was purified on Protein-A affinity columns followed by dialysis into phosphate-buffered saline.

### Capture and detection of HIV with nanoparticles

MNP-anti-gp120 antibody complexes were labeled with goat-anti human Fab fragments (Zenon Human Antibody Labeling Kit, Invitrogen). We separated unbound free Fab fragments from ones associated with MNP-anti-gp120 complexes by washing twice on 100 K Nanosep centrifugal device. Labeled MNP-Ab complexes (3.9 × 10^12^ particles in 60 µl) were incubated at 37 °C for 40 minutes (continuous mixing) with HIV viral stocks (~8 × 10^6^ in 60 µl)^14^. Various combinations of detection fluorescent antibodies (PG9, PG16, 2G12, 4B3 (Polymun Scientific), b6 (a kind gift from Dr. Pascal Poignard), and VRC01 (a kind gift from Dr. John Mascola)) were added to the mixture and incubated at room temperature for 20 minutes. HIV virions captured with MNPs and stained with detection antibodies were separated from non captured virions and unbound fluorescent detection antibodies on MACS magnetic columns (Miltenyi Biotech, Auburn, CA) attached to a high field MACS magnet (Miltenyi Biotech). After washing columns three times with washing buffer (0.5% BSA, 1 mM EDTA), we removed them from the magnet, eluted demagnetized MNPs-HIV-Ab complexes from the column in PBS and fixed with 1% PFA.

### Analysis of HIV virions with flow cytometer

Purified complexes were analyzed with an LSRII (BD Biosciences, San Jose, CA) flow cytometer equipped with 355-, 407-, 488-, 532- and 638-nm lasers and equipped with inline filter with a nominal cutoff of 40 nm (Meissner, Camarillo, CA). The background level of fluorescence was evaluated with 0.1-µm filtered PBS. Data were acquired in a fixed volume of 160 *μ*L in duplicate using a High Throughput Sampler (HTS) at a flow rate of 0.5 *μ*L per second on the LSRII flow cytometer. The threshold was set up to the lowest fluorescence channel that did not generate an AlexaFluor 488/AlexaFluor 594 signal with this solution. The samples were acquired in the volume of ~200 *μ*L. Data were acquired with Diva 6.3 and were analyzed with FlowJo software v9.4.9 (Treestar Software, Ashburn, OR).

### Depletion of HIV viral stocks

PG16-, 4B3-, VRC01 or mouse IgG (used as control)- coupled MNPs (1.3 × 10^13^ particles) were added to HIV viral stock (15 ng in 200 µl) and incubated at 37 °C for 1 hour. Next, HIV virions captured by MNPs were passed through MACS magnetic columns. Virions captured by specific MNPs were retained on the column and non-captured virions were collected in culture medium as depleted fraction and used for further infection of tonsillar tissue *ex*-*vivo*.

### Human *ex vivo* tissues

Human tonsillar tissues were obtained from routine tonsillectomy (unrelated to the current study) performed in the Children’s Hospital (Washington, DC). Tissues were received from the Pathology Department and were considered as “pathological waste”. Tissue samples were anonymized and the protocol was approved by the NIH Office of Human Subject Research. Human tonsillar tissues were dissected into small blocks (27 tissue blocks per condition at 9 blocks per well) and placed onto collagen sponge gels in culture medium at the air-liquid interface as described earlier^26^. Each experimental condition was infected with HIV-I_BaL_ depleted of virions recognized by either PG16, 4B3, or VRC01. In addition a control for nonspecific binding viral stock was incubated with mouse IgG-MNPs. Replication of HIV was evaluated by release of p24 into culture medium^38^.

### Statistical analysis

All statistical analyses were performed with JMP 11.0 (SAS Institute, Cary, NC). Data normality was verified by the Shapiro-Wilk W test. Groups with normally distributed parameters were compared using Paired t-Student’s test.

## Electronic supplementary material

Supplementary Info File 1
