# Supplementary material for: Flow virometry analysis of envelope glycoprotein conformations on individual HIV virions
Source: Sci Rep. 2017 Apr 19;7:948. doi: 10.1038/s41598-017-00935-w (PMC5430429; doi:10.1038/s41598-017-00935-w)
Supplement: Supplementary file 1 — Supplementary Info File 1 [file 41598_2017_935_MOESM1_ESM.pdf]

Supplementary material

Flow virometry analysis of envelope glycoprotein conformations on individual HIV virions

Anush Arakelyan[1], Wendy Fitzgerald[1], Deborah F. King[2], Paul Rogers[2], Hannah M. Cheeseman[2], Jean-Charles Grivel[1][illegible],
Robin J. Shattock[2] and Leonid Margolis[1]

[1]Section of Intercellular Interactions, Eunice Kennedy Shriver National Institute of Child Health and Human Development, National Institutes of Health, Bethesda, Maryland and [2]Mucosal Infection & Immunity Group, Department of Medicine, Imperial College, London, UK.

[illegible]

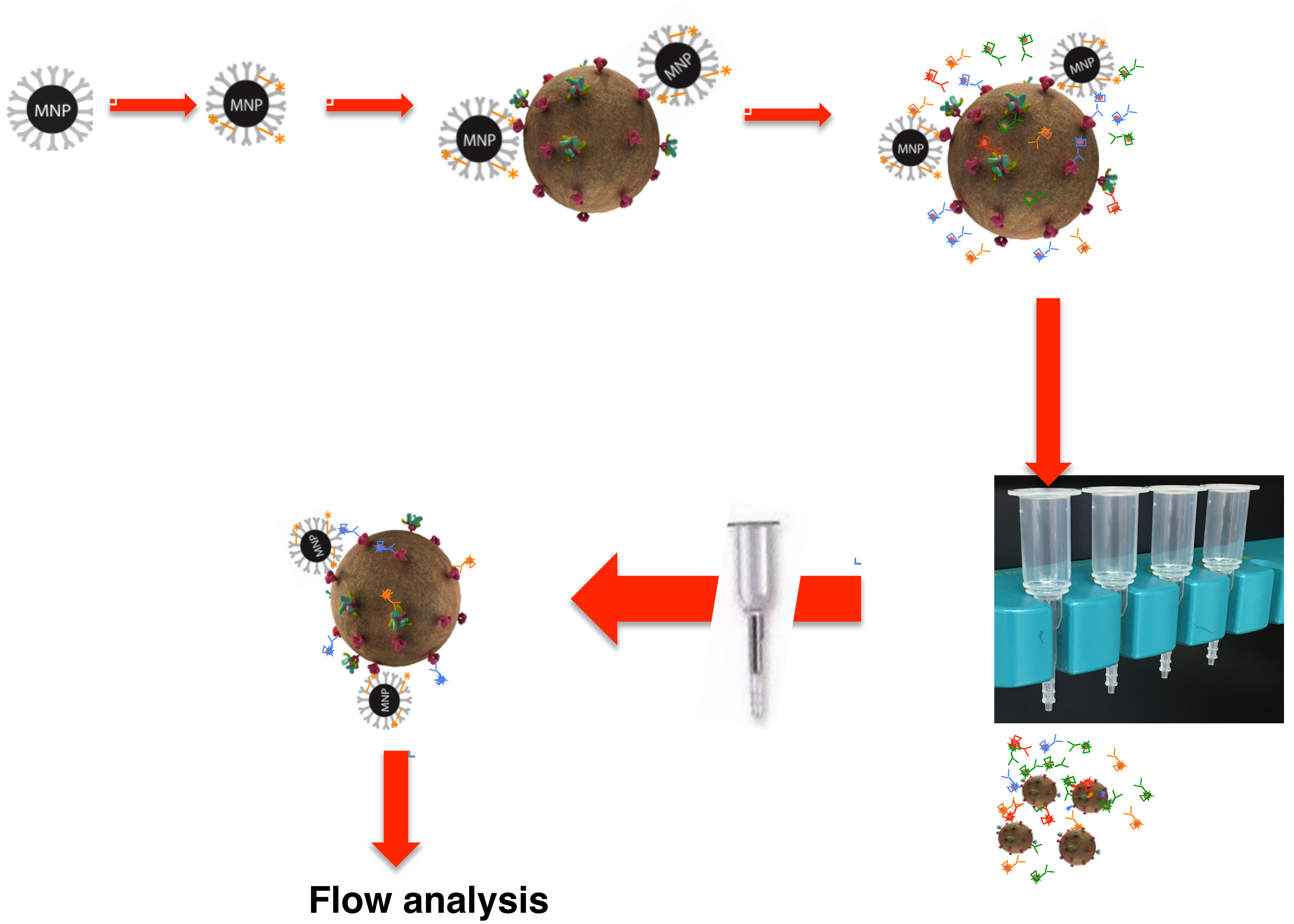

Figure S1. Principle ŧĨow virometry of individual HIV [illegible]
Magnetic nanoparticles (MNPs) coupled with anti-HIV antibodies were stained with fluorescent anti-human IgG1 Fab fragments. HIV virions were captured with antibody-coupled MNPs and the resultant complexes stained with fluorescent monoclonal antibodies against antigens of interest were separated from free antibodies and non-captured virions in a strong magnetic field using magnetic columns. The eluted complexes were analyzed with a flow cytometer triggered on fluorescence.

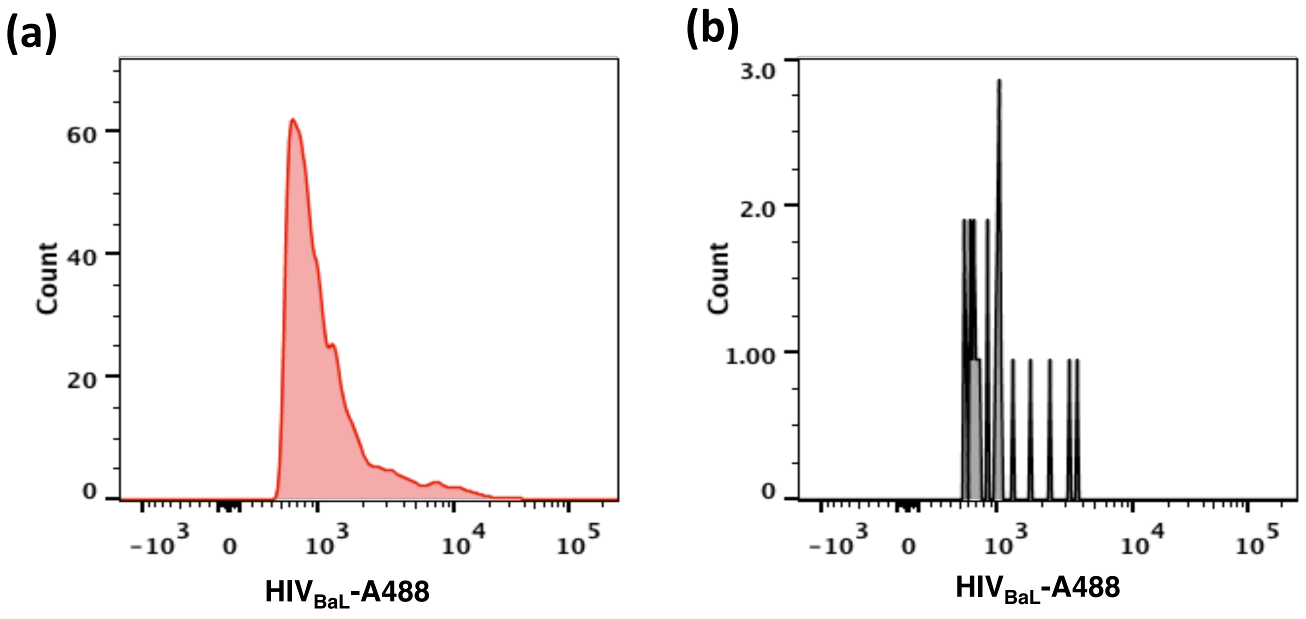

Figure S2. Efficiency of the MNP capture assay

AlexaFluor 488-labeled HIV-1_{BaL} was captured with 2G12-MNPs, isolated on a magnetic column, and analyzed with a flow cytometer (**a**). The non-captured (flow-through) fraction was captured again with 2G12-MNPs, isolated on a magnetic column, and analyzed on a flow cytometer (**b**). A representative experiment out of three is shown. 95% of virions were captured and analyzed in the first run.

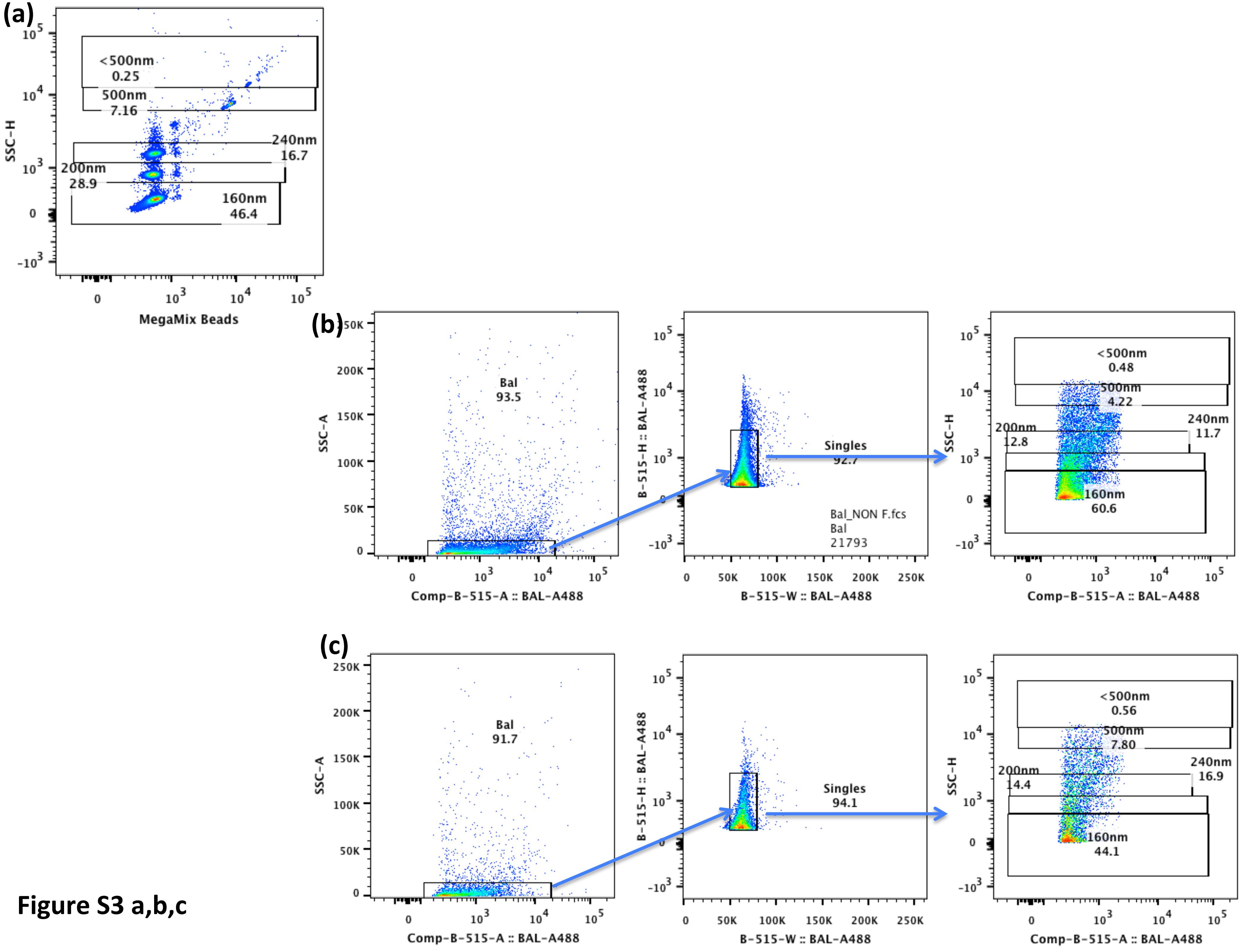

Figure S3 a,b,c

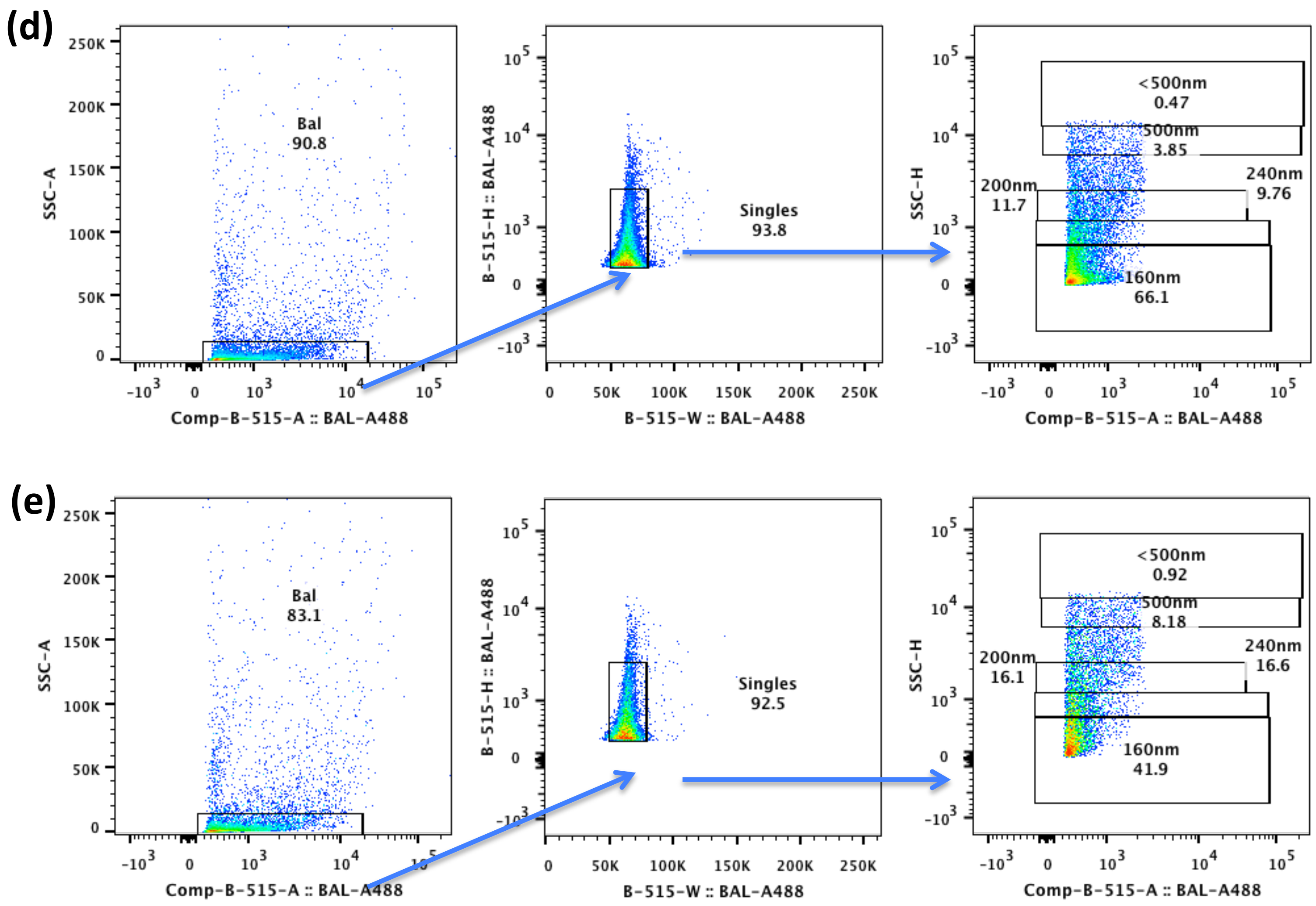

Figure S3. Sizing of the MNP captured HIV

(a) Calibrating beads of different size. (**b)** AlexaFluor 488-labeled HIV-1$_{BaL}$ captured with 2G12-MNPs, isolated on a magnetic column, and analyzed with a flow cytometer.**(c)** AlexaFluor 488-labeled HIV-1$_{BaL}$ was filtered through a 0.22µm filter, captured with 2G12-MNPs, isolated on a magnetic column, and analyzed with a flow cytometer. **(d)** AlexaFluor 488-labeled HIV-1$_{BaL}$ captured with 2G12-MNPs stained with PG9 antibodies, isolated on a magnetic column, and analyzed with a flow cytometer. **(e)** AlexaFluor 488-labeled HIV-1$_{BaL}$ was filtered through 0.22µm filter, captured with 2G12-MNPs, stained with PG9 antibodies, isolated on a magnetic column, and analyzed with a flow cytometer.

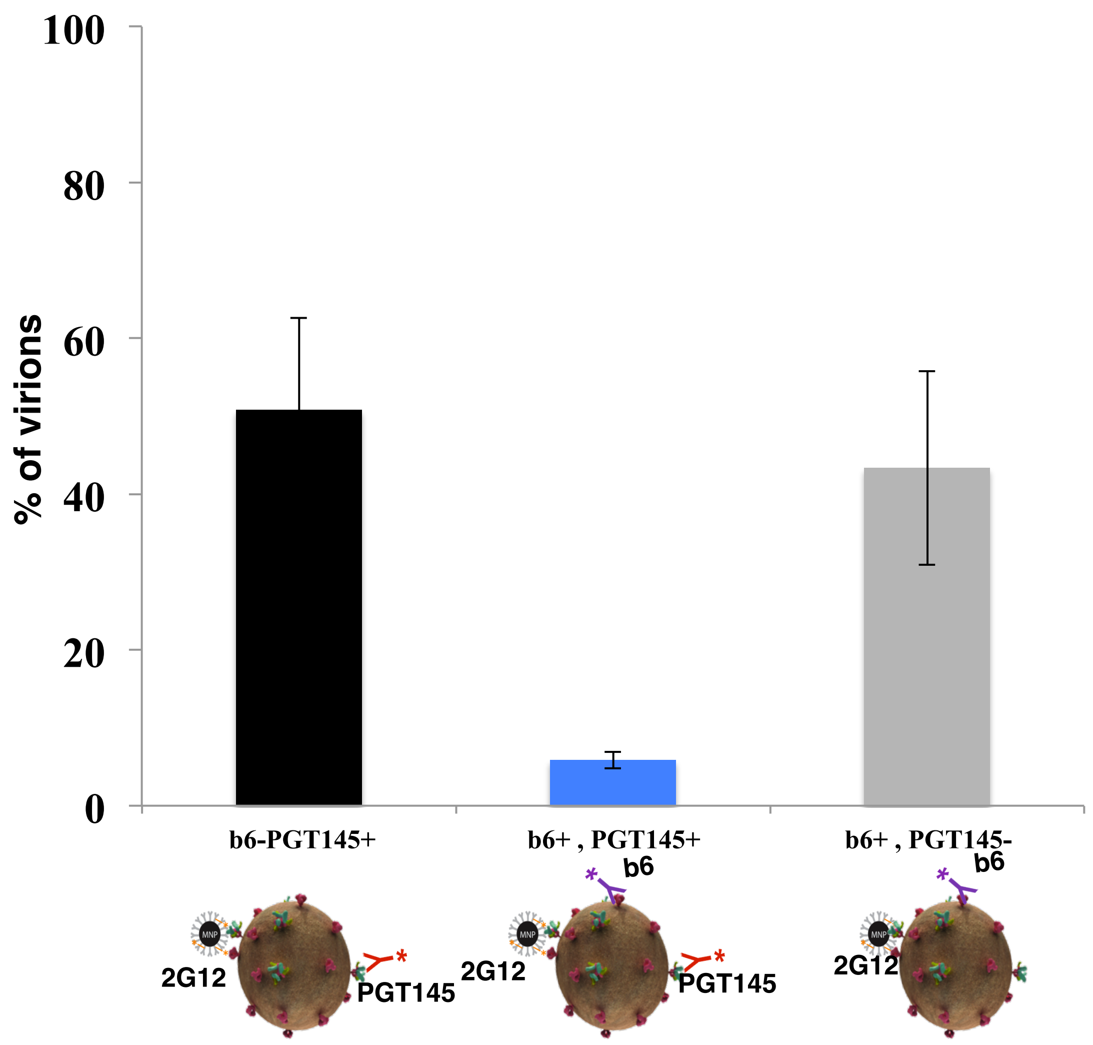

Figure S4. Flow virometry of HIV-1$_{BaL}$ virions captured with 2G12-MNPs

Distribution of 2G12-captured virions positive for PGT145 and/or for b6. Each bar represents mean ± SEM of three experiments.

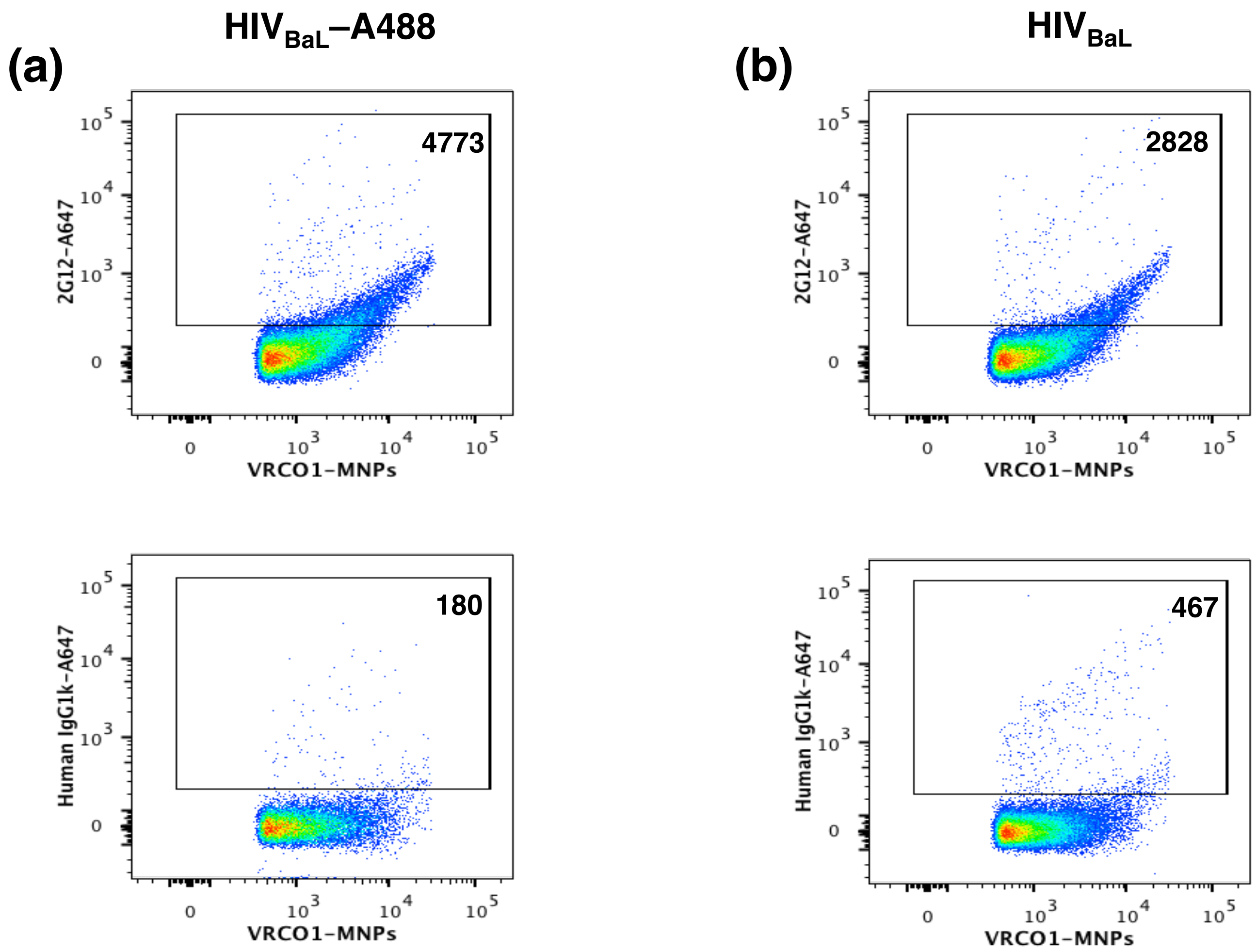

Figure S5. Comparison of Alexa Fluor -labeled and unlabeled HIV-1$_{BaL}$ virions
Virions labeled with Alexa Fluor 488 as described in Methods **(a)** or non-labeled virions **(b)** were captured with VRC01-MNPs and revealed with AlexaFluor 647-labeled 2G12 antibodies (upper panels) or with the isotype control AlexaFluor 647-labeled antibodies (lower panels).

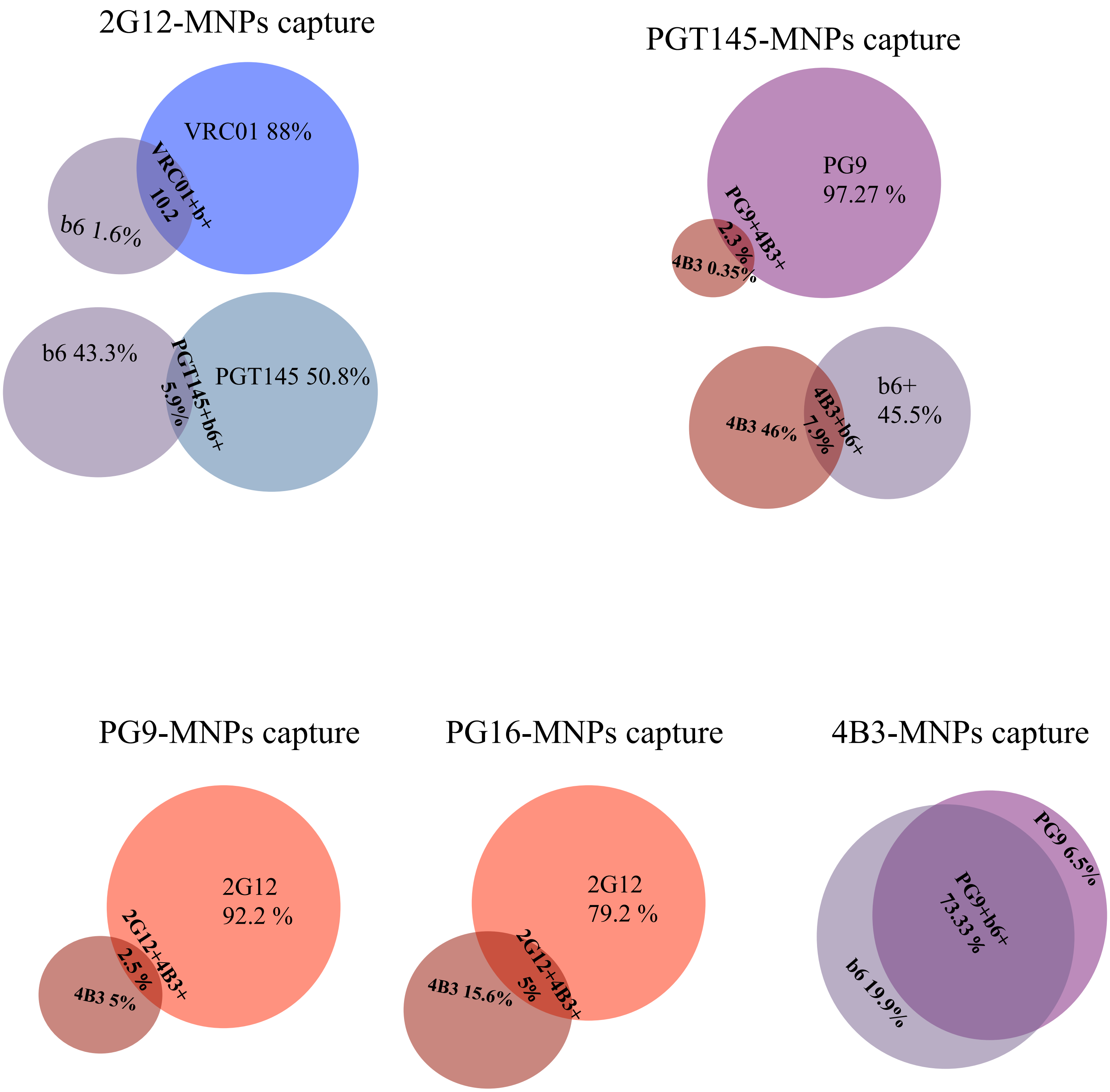

Figure S6. Venn Diagrams of HIV virions carrying various Env conformations

Table S1. Capture and detection antibodies

In the left column of the table shown are capture antibodies. In the two right columns shown are detection antibodies.

2G12 recognize clusters of high mannose type glycans on the outer domain of gp120[18,23]; VRC01 preferentially recognizes the CD4 binding site (CD4bs) on all gp120[19]; b6 preferentially recognizes the CD4bs[20,22] on uncleaved gp160 as well as non-trimeric forms of gp120; PGT145 binds to quaternary epitope at the V2 apex; PG9 and PG16[21] preferentially recognize quaternary epitopes that are trimer- and glycan-dependent and which bind to the V2 apex; 4B3 recognizes cluster I on gp41[17]; PGT151 recognizes quaternary, cleavage-dependent epitope at the gp41-gp120 interface[24].

MNPs	Detection Abs	
2G12	VRCO1	b6
2G12	PGT145	b6
PGT145	PG9	4B3
PGT145	b6	4B3
PG9	2G12	4B3
PG16	2G12	4B3
4B3	PG9	b6
VRC01	PGT151	b6, 4B3
PGT145	PGT151	b6, 4B3

Pairs of detection antibodies	Competition Coefficient (CC)
VRC01/b6	[VRC01] 1.3±0.1*(n=2)*<br>[b6] 1.6±0.1*(n=2)*
PG9/4B3	[PG9] 1.6±0.3*(n=3)*<br>[4B3] 1.2±0.6*(n=3)*
PGT145/b6	[PGT145] 1.3±0.3*(n=3)*<br>[b6] 1.0±0.3*(n=3)*
PGT145/4B3	[PGT145] 0.8±0.08*(n=2)*<br>[4B3] 1.4±0.01*(n=2)*
4B3/b6	[4B3] 1.6±0.3*(n=3)*<br>[b6] 1.1±0.3*(n=3)*
PG9/PGT145	[PGT145] 1.0±0.2*(n=2)*<br>[PG9] 0.4±0.02*(n=2)*

Table S2. To check the possible competition between different detection antibodies used in the present work we captured virions with MNPs coupled to 2G12 (that have a broad recognition pattern) and stained with various detection antibodies used either alone or in combinations. As a positive control we tested PG9 and PGT145 ǿKǐ0K ōǐyŘ to closely –situated epitopes.

Presented in the table are competition coefficients (CC) calculateŘ as N_{al}/N_{comb} where N_{al} is the number of positive events when a particular antibody was used alone and N_{comb} the number of positive events when this antibody was used in combination with the second one.

CC=1 indicates no competition between two antibodies. Presented are means ± SEM of replicates or triplicateǎ for each experimental condition.

Note that a combination of PGT145 and PG9 antibodies was not used for detection in the present work but presented as a positive control for the competition assay since these antibodies bind to very closely-situated epitopes on Env .
